# Supplementary material for: Changes in seam number and location induce holes within microtubules assembled from porcine brain tubulin and in Xenopus egg cytoplasmic extracts
Source: eLife. 2022 Dec 12;11:e83021. doi: 10.7554/eLife.83021 (PMC9788831; doi:10.7554/eLife.83021)
Supplement: Supplementary file 1. — Sub-tomogram averages and their corresponding models can be opened in Imod using the following command: 3dmod -V -E U emd_XXXX.map EMD-XXXX_helix.mod. [file elife-83021-supp1.docx]

**Supplementary Table 1. EMDB deposition IDs**

| **IDs** | **Figure - Video** | **Helix model** |
| --- | --- | --- |
| EMD-15735 | Fig. 2A - Figure 2—Video 2 | EMD-15735_helix.mod |
| EMD-15736 | Fig. 3A - Figure 3—Video 3 | EMD-15736_helix.mod |
| EMD-15737 | Fig. 3B - Figure 3—Video 3 | EMD-15737_helix.mod |
| EMD-15738 | Fig. 3B - Figure 3—Video 3 | EMD-15738_helix.mod |
| EMD-15739 | Fig. 3B - Figure 3—Video 3 | EMD-15739_helix.mod |
| EMD-15734 | Fig. 4A | - |
| EMD-15740 | Fig. 5A | EMD-15740_helix.mod |
| EMD-15741 | Fig. 5A | EMD-15741_helix.mod |
| EMD-15742 | Fig. 5B | - |
| EMD-15743 | Fig. 6B | - |
| EMD-15750 | Fig. 6A | - |
| EMD-15751 | Fig. 6D | - |
| EMD-15752 | Fig. 6C | - |
| EMD-15732 | Fig. 7C | - |
| EMD-15733 | Fig. 8A - Figure 8—Video 5 | EMD-15733_helix.mod |
| EMD-15744 | Fig. 8A - Figure 8—Video 5 | EMD-15744_helix.mod |
| EMD-15745 | Fig. 8A - Figure 8—Video 5 | EMD-1574_helix.mod |
| EMD-15746 | Fig. 9A - Figure 9—Video 6 | EMD-15746_helix.mod |
| EMD-15747 | Fig. 9B - Figure 9—Video 6 | EMD-15747_helix.mod |
| EMD-15748 | Fig. 9C - Figure 9—Video 6 | EMD-15748_helix.mod |
| EMD-15749 | Fig. 9D - Figure 9—Video 6 | EMD-15749_helix.mod |

Sub-tomogram averages and their corresponding models can be opened in Imod using the following command: 3dmod -V -E U emd_XXXX.map EMD-XXXX_helix.mod.
