# Supplementary material for: Changes in seam number and location induce holes within microtubules assembled from porcine brain tubulin and in Xenopus egg cytoplasmic extracts
Source: eLife. 2022 Dec 12;11:e83021. doi: 10.7554/eLife.83021 (PMC9788831; doi:10.7554/eLife.83021)
Supplement: Supplementary file 2. — To reconstruct the volumes, follow the instructions in the SSTA.txt document. [file elife-83021-supp2.docx]

**Supplementary Table 2. EMPIAR deposition IDs**

| IDs | Sample | Raw tilt series | Reconstructed tomograms | Models |
| --- | --- | --- | --- | --- |
| EMPIAR-11253 | GTP sample 1 | GTP_S1_tomo1 | GTP_S1_tomo1.rec | MT1 - MT2 |
|  |  | GTP_S1_tomo2 | GTP_S1_tomo2.rec | MT3 - MT6, MT8 - MT10 |
|  | GTP sample 2 | GTP_S2_tomo1 | GTP_S2_tomo1.rec | MT7, MT11 - MT17 |
|  |  | GTP_S2_tomo2 | GTP_S2_tomo2.rec | MT18 - MT24 |
| EMPIAR-11263 | Xenopus-DMSO | Xen_D_tomo1 | Xen_D_tomo1_rec.mrc | MT1 - MT16 |
|  |  | Xen_D_tomo2 | Xen_D_tomo2_rec.mrc | MT13 - MT20 |
|  |  | Xen_D_tomo3 | Xen_D_tomo3_rec.mrc | MT21 - MT28 |
|  |  | Xen_D_tomo4 | Xen_D_tomo4_rec.mrc | MT29 - MT43 |
|  |  | Xen_D_tomo5 | Xen_D_tomo5_rec.mrc | MT44 - MT64 |
|  | Xenopus-RanQ69L | Xen_Ran_tomo1 | Xen_Ran_tomo1_rec.mrc | MT1 - MT15 |
| EMPIAR-11264 | GMPCPP sample 1 | GMPCPP_S1_tomo1 | GMPCPP_S1_tomo1_rec.mrc | MT1 - MT7 |
|  |  | GMPCPP_S1_tomo2 | GMPCPP_S1_tomo2_rec.mrc | MT15 |
|  |  | GMPCPP_S1_tomo3 | GMPCPP_S1_tomo3_rec.mrc | MT8 - MT12 |
|  |  | GMPCPP_S1_tomo4 | GMPCPP_S1_tomo4_rec.mrc | MT13 - MT14 |
|  | GMPCPP sample 2 | GMPCPP_S2_tomo1 | GMPCPP_S2_tomo1_rec_bin2.mrc | MT21 - MT31 |
|  |  | GMPCPP_S2_tomo2 | GMPCPP_S2_tomo2_rec_bin2.mrc | MT16 - MT20 |
|  | GMPCPP_tomoFig5 | GMPCPP_tomoFig5 | GMPCPP_tomoFig5_bin4.mrc | GMPCPP_segment1_Fig5  GMPCPP_segment2_Fig5 |
|  | GMPCPP_tomoFig6 | GMPCPP_tomoFig6 | GMPCPP_tomoFig6_bin4.mrc | GMPCPP_tomoFig6B  GMPCPP_tomoFig6D |

To reconstruct the volumes, follow the instructions in the SSTA.txt document.
